# Deep learning with diffusion basis spectrum imaging for classification of multiple sclerosis lesions

**DOI:** 10.1002/acn3.51037

**Published:** 2020-04-18

**Authors:** Zezhong Ye, Ajit George, Anthony T. Wu, Xuan Niu, Joshua Lin, Gautam Adusumilli, Robert T. Naismith, Anne H. Cross, Peng Sun, Sheng‐Kwei Song

**Affiliations:** ^1^ Department of Radiology Washington University School of Medicine St. Louis Missouri 63110; ^2^ Department of Biomedical Engineering Washington University St. Louis Missouri 63130; ^3^ Keck School of Medicine University of Southern California Los Angeles California 90033; ^4^ Department of Neurology Washington University School of Medicine St. Louis Missouri 63110

## Abstract

**Objective:**

Multiple sclerosis (MS) lesions are heterogeneous with regard to inflammation, demyelination, axonal injury, and neuronal loss. We previously developed a diffusion basis spectrum imaging (DBSI) technique to better address MS lesion heterogeneity. We hypothesized that the profiles of multiple DBSI metrics can identify lesion‐defining patterns. Here we test this hypothesis by combining a deep learning algorithm using deep neural network (DNN) with DBSI and other imaging methods.

**Methods:**

Thirty‐eight MS patients were scanned with diffusion‐weighted imaging, magnetization transfer imaging, and standard conventional MRI sequences (cMRI). A total of 499 regions of interest were identified on standard MRI and labeled as persistent black holes (PBH), persistent gray holes (PGH), acute black holes (ABH), acute gray holes (AGH), nonblack or gray holes (NBH), and normal appearing white matter (NAWM). DBSI, diffusion tensor imaging (DTI), and magnetization transfer ratio (MTR) were applied to the 43,261 imaging voxels extracted from these ROIs. The optimized DNN with 10 fully connected hidden layers was trained using the imaging metrics of the lesion subtypes and NAWM.

**Results:**

Concordance, sensitivity, specificity, and accuracy were determined for the different imaging methods. DBSI‐DNN derived lesion classification achieved 93.4% overall concordance with predetermined lesion types, compared with 80.2% for DTI‐DNN model, 78.3% for MTR‐DNN model, and 74.2% for cMRI‐DNN model. DBSI‐DNN also produced the highest specificity, sensitivity, and accuracy.

**Conclusions:**

DBSI‐DNN improves the classification of different MS lesion subtypes, which could aid clinical decision making. The efficacy and efficiency of DBSI‐DNN shows great promise for clinical applications in automatic MS lesion detection and classification.

## Introduction

Multiple sclerosis (MS) is a common inflammatory central nervous system (CNS) disorder that affects over 600,000 people in the United States.^1^ MS usually begins with intermittent “attacks” (i.e., relapsing‐remitting course) characterized by transient episodes of CNS dysfunction.^2^ These clinical attacks, or relapses, are caused by focal inflammation in the CNS.^3^ Once the acute inflammation subsides, the acute lesions become chronic, and may be characterized by varying degrees of demyelination, axonal injury and loss, gliosis, and residual inflammation.^3^

Conventional MRI (cMRI) is often used to characterize and quantify MS lesions in the CNS,^4^ with lesion subtypes being identified and classified based on their intensity using from MR sequences.^5^ Hypointense areas of white matter (WM) on T1‐weighted imagin***g*** (T1WI) are commonly known as “black holes” (BHs) and “gray holes” (GHs), depending upon the level of hypointensity. BHs and GHs persisting for at least 12 months are markers of focal tissue injury in MS and are known as “persistent black holes” (PBHs) and “persistent gray holes” (PGHs).^6^ Based on histological correlations, PBH are considered to contain more severe axonal loss compared with other MS lesion subtypes^7^. Other MS lesions that are hyperintense on T2‐weighted imaging (T2WI) and lack hypointensity on T1WI have less severe tissue damage, and are referred to here as nonblack or gray hole (NBH) lesions.^8^, ^9^ While standard cMRI is sensitive in detecting MS lesions in WM, it requires experience to categorize the lesion subtype, in addition to longitudinal follow‐up.^10^

Our laboratory developed a novel diffusion basis spectrum imaging (DBSI) method,^11^, ^12^ and demonstrated its ability to quantitatively characterize the pathologies that underlie MRI lesions in a biopsy of a demyelinating brain lesion and in postmortem MS specimens.^13^, ^14^ While DBSI‐derived metrics were correlated with axonal injury/loss, demyelination, and inflammation,^11^, ^15^ a comprehensive analysis employing DBSI‐derived metrics to detect and differentiate cMRI‐based MS lesion subtypes have yet to be conducted. Herein, we introduce a novel imaging approach which combines DBSI‐derived structural metrics (as the classifiers) with a deep neural network (DNN) algorithm. We tested the performance of DBSI‐DNN in detecting and classifying the various MS lesion subtypes, and compared it to cMRI, as well as DTI and MTR.

## Materials and Methods

### Subject

The study was approved by the Institutional Review Board of Washington University School of Medicine. Thirty‐eight people with MS were enrolled after providing written informed consent. Patient information and details are included in Table 1.

**Table 1:** Patient and lesion characteristics.

Patient/lesion characteristics	No.
Patient	38
Age (years), median (range)	55 (25–72)
Disease duration (years), median (range)	13.7 (1.5–43.3)
EDSS, median (range)	6 (1.5–6.5)
Gender (male/female)	12/26
MS subtypes	
RRMS	13
PPMS	15
SPMS	10
MS lesion types	
PBH	92
PGH	89
AGH	16
NBH	189
NAWM	113

### Image acquisition

Patients were imaged on a 3.0‐T Siemens Trio scanner (Siemens, Erlangen, Germany). T1WI was acquired using the following parameters: Repetition Time (TR) = 600 ms; Echo Time (TE) = 9 ms; slice thickness = 2 mm; in‐plane resolution = 1 × 1 mm^2^; total acquisition time = 4 min. Magnetization‐prepared rapid gradient‐echo (MPRAGE) image with isotropic 1 mm^3^ resolution was used for identification of structural landmarks and as a registration target (TR = 2400 ms, TE = 3.16 ms, TI = 1000 ms, FOV = 256 × 224 mm^2^). T2WI using fluid‐attenuated inversion recovery sequence was acquired to quantify visible WM lesion volumes (TR = 7500 ms, TI = 2500 ms, TE = 210 ms, FOV = 256 × 256 mm^2^, Resolution = 1 × 1 × 1 mm^3^). Magnetization transfer (MT) images were acquired with the following parameters: TR = 43 ms; TE = 11 ms; Flip Angle = 30 degrees; FOV = 192 × 256 mm^2^; slice thickness = 3 mm; in‐plane resolution = 1 × 1 mm^3^; total acquisition time = 8 min. Magnetization transfer ratio (MTR) maps were calculated pixel‐by‐pixel using the equation: MTR = (S_off _– S_on_)/S_off_ × 100, where S_on_ and S_off_ were signal intensities with and without saturation pulse. Axial diffusion‐weighted images (DWI) covering the whole brain were acquired using a multi‐b value diffusion weighting scheme (99 directions, maximum b‐value 1500 s/mm^2^) and the following parameters: TR = 10,000 ms; TE = 120 ms; FOV = 256 × 256 mm^2^; slice thickness = 2 mm; in‐plane resolution = 2 × 2 mm^2^; total acquisition time = 15 min. Eddy current and motion artifacts of DWI were corrected before susceptibility‐induced off‐resonance field was estimated and corrected.^16^

### MS lesion identification

**Figure 1:**
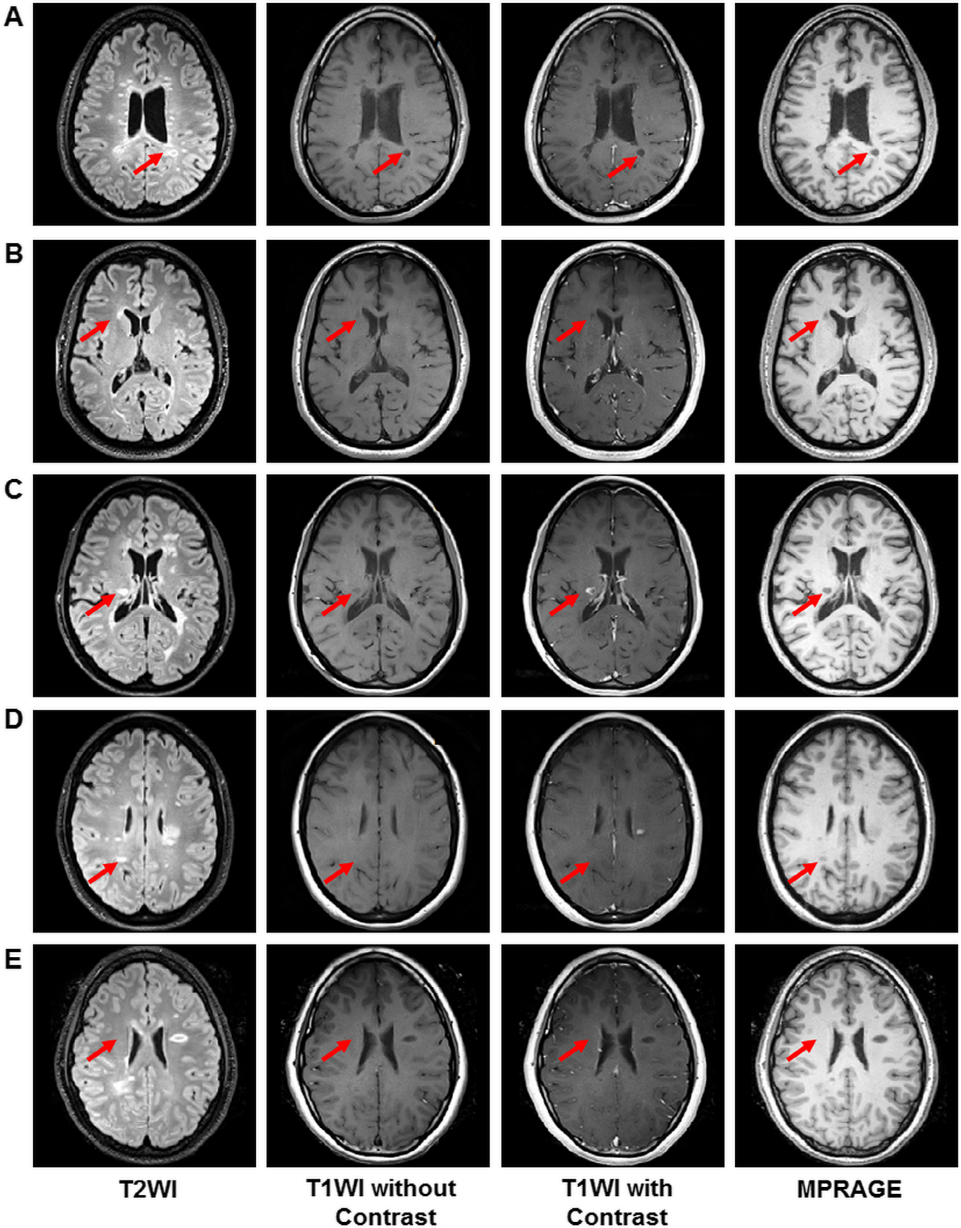
Different MS lesion subtypes. Columns from left through right are representative cases of persistent black hole (PBH) lesion (A), persistent gray hole (PGH) lesion (B), acute gray hole (AGH) lesion (C), nonblack or gray hole (NBH) lesion (D), and normal appearing white matter (NAWM) region (E). Red arrows indicated the location of MS lesions.

Lesions were classified as being black hole, gray hole, or nonblack or gray hole using an objective and semiquantitative intensity ratio (IR) method developed in our previous work^9^. This method uses a protocol that allows a single investigator to reliably determine lesion types. The range of IR used for black hole, gray hole, and nonblack or gray hole were 1.00 to 1.70, 1.71 to 2.60, and > 2.60, respectively ^9^. PBHs (Fig. 1A) and PGHs (Fig. 1B) were defined as being present for at least 12 months, and not in the setting of contrast enhancement. Acute black hole (ABH) and acute gray hole (AGH) were hypointensities on T1WI within currently contrast enhancing lesions (Fig. 1C). ABHs were not included in this study because the number of ABHs identified was insufficient for model training. Nonblack or gray hole (NBH, Fig 1D) lesions were hyperintense on T2WI images without hypointensity on T1WI, and had IR> 2.60. NAWM ROIs were delineated from the contralateral side of the brain to the lesions, in areas with no lesions or “dirty‐appearing” white matter on T2WI imaging (Fig. 1E). NAWM ROI volumes were purposely made comparable to lesion volumes to avoid class imbalances.

All the lesions in this manuscript were classified by the IR method, used by a neurologist with> 20 years of clinical experience prior to being analyzed in this study. Amira 6.0.1 visualization and analysis software (FEI, Hillsboro, OR) was used to quantify intensity for each hypointense lesion on all scans. Note that the lesion intensity assessment requires establishing the baseline intensity of each scan to account for scan‐to‐scan intensity variations.

### Diffusion basis spectrum imaging

DBSI models the diffusion‐weighted MRI signals as a linear combination of multiple tensors describing both the discrete anisotropic content (axonal fibers) and an isotropic diffusion spectrum component encompassing the full range of diffusivities, Eq. [1].^12^

(1) $$S_{k}=\sum_{i=1}^{N_{\mathrm{Aniso}}}f_{i}e^{-\left|\vec{b_{k}}\right|\cdot \lambda_{⊥\_i}}e^{-\left|\vec{b_{k}}\right|\cdot \left(\lambda_{{\|}_{i}}-\lambda_{{⊥}_{i}}\right)\cdot \cos^{2}\psi_{\mathrm{ik}}}+\int_{a}^{b}f\left(D\right)e^{-\left|\vec{b_{k}}\right|\cdot D}dD\left(k=1,2,\ldots ,K\right)$$

where S*_k_* and $\left|b_{k}\right|$ are the normalized signal and b‐value of the k^th^ diffusion gradient, *N_Aniso_* is the number of anisotropic tensors, $\psi_{\mathrm{ik}}$ is the angle between the k^th^ diffusion gradient and the principal direction of the *i^th^* anisotropic tensor, $\lambda_{{\|}_{i}}$ and $\lambda_{{⊥}_{i}}$ are the axial diffusivity (AD) and radial diffusivity (RD) of the *i^th^* anisotropic tensor, *f_i_* is the signal intensity fraction for the *i^th^* anisotropic tensor, and *a* and *b* are the low and high diffusivity limits for the isotropic diffusion spectrum *f(D)*. The anisotropic diffusion component describes water molecules inside and outside myelinated or nonmyelinated axons. DBSI‐derived anisotropic signal fractions (*f_i_*, i.e., fiber fraction) reflects the apparent axonal density in WM. DBSI‐derived AD and RD retain the pathological specificity for axon and myelin integrity as in previously published models,^17^, ^18^ without confounds from non–fiber related changes. The DBSI‐derived “restricted” isotropic diffusion fraction (ADC ≤ 0.3 µm^2^/ms) has been shown to reflect cellularity.^12^ Hindered (0.3 µm^2^/ms ≤ ADC ≤3 µm^2^/ms) and free (ADC ≥ 3 µm^2^/ms) isotropic diffusion components represent water molecules in less densely packed environments, such as areas of tissue disintegration or edema, or contaminating cerebrospinal fluid (CSF).^12^, ^19^, ^20^

### Image processing

Whole‐brain voxel‐wise DTI and DBSI analyses were performed by an in‐house software developed using MATLAB^®^ (MathWorks). To control for scan‐to‐scan variation within individual scans, cerebrospinal fluid (CSF), which is unaffected by MS pathologies, was used as the baseline for individual scans and to assess signal intensities of MS lesions on T1WI and T2WI. Regions of CSF (≥ 100 voxels) were defined on axial slices where the anterior horns of the lateral ventricles were widest. Voxels containing choroid plexus or within two voxel distance from the ventricle edge were excluded. For each voxel in MS lesions, the voxel intensity was divided by the CSF intensity to normalize b0, T1WI, and T2WI intensities.

### DNN model development and optimization

**Figure 2:**
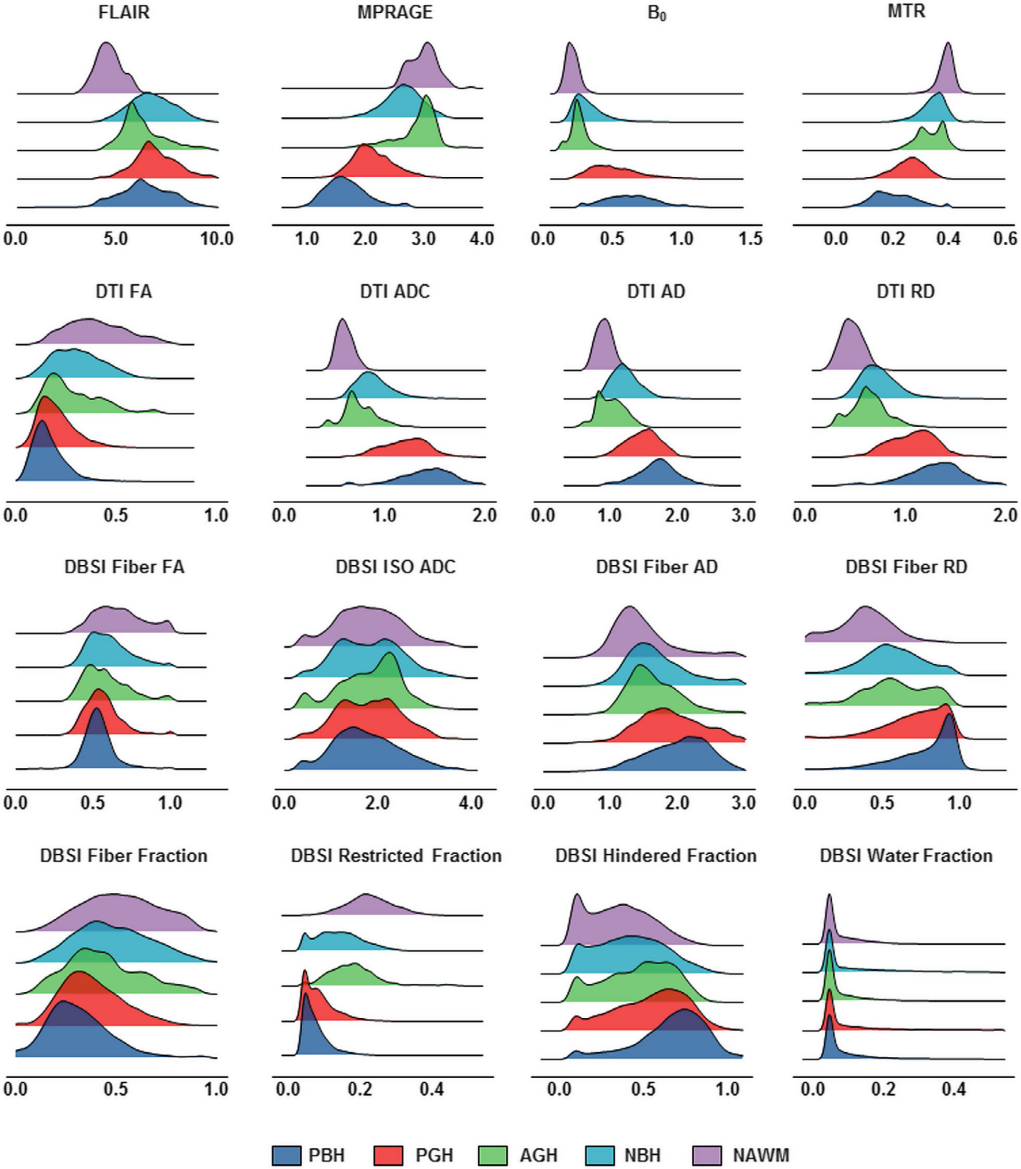
Distribution histograms of different MRI metrics for different MS lesion types. Imaging voxels from persistent black holes (PBH, dark blue), persistent gray holes (PGH, red), acute gray holes (AGH, green), nonblack or gray hole lesions (NBH, cyan), and normal appearing white matter (NAWM, purple) were plotted to show the distributions for cMRI, MTR, DTI, and DBSI metrics. X‐axis = metric intensities; Y‐axis = frequency; ADC = apparent diffusion coefficient; FA = fractional anisotropy; AD = axial diffusivity; RD = radial diffusivity.

Our complete dataset consisted of 43,261 imaging voxels from 499 MS lesions obtained from 38 patients. The collected voxels were split into training, validation, and test datasets with a ratio of 8:1:1, respectively. Imaging voxels from test datasets were separated from lesions that were used in the training and validation steps. Validation set was employed to fine tune the model hyper‐parameters. We then compared four different DNN models that incorporate different MRI metrics. The first DNN model (DBSI‐DNN) incorporated both DBSI metrics and normalized T1WI and T2WI intensities. The second model (DTI‐DNN) used DTI metrics in conjunction with normalized T1WI and T2WI intensities. The third model (MTR‐DNN) used MTR metric in combination with normalized T1WI and T2WI intensities. The fourth DNN model used normalized T1WI and T2WI intensities from cMRI alone. The diffusion metrics assessed with our DNN modeling included eight diffusion metrics provided from DBSI and another four from DTI (Fig. 2). Specifically, DBSI metrics include, fiber fraction, fiber fractional anisotropy (FA), fiber AD, fiber RD, restricted isotropic diffusion fraction (restricted fraction), hindered isotropic diffusion fraction (hindered fraction), free isotropic diffusion fraction (water fraction) and normalized b0 intensity. DTI metrics include ADC, FA, AD, and RD.

The DNN model was developed using Tensorflow frameworks in Python.^21^ In general, the DNN models were constructed with multiple fully connected neural network. Exponential linear units (ELU) were used to activate specific functions in each hidden layer. Batch normalization was performed with a mini‐batch size of 200 before feeding data to the next hidden layer to improve model optimization and to prevent overfitting. The final layer was a fully connected softmax layer that produces a likelihood distribution over the five output classes. DNN models with varying numbers of hidden layers, nodes and training epochs were tested for model optimization. The network was trained with random initialization of the weights as described.^22^ The Adam optimizer was used with the default parameters of β_1_ = 0.9 and β_2_ = 0.999 and a mini‐batch size of 200. The cross‐entropy loss function was chosen, and the model was trained to minimize the error rate on the development dataset. Generally, the hyper‐parameters of the network architecture and optimization algorithm were chosen through a combination of grid search and manual tuning.

### Statistical analysis

Confusion matrices were calculated and used to illustrate the specific examples of MS lesion classes where the DNN prediction contradicts the neurologist’s diagnoses. The one‐versus‐rest strategy was implemented to perform ROC analysis; and area under curve (AUC) was calculated to assess model discrimination of each lesion type. Sensitivity and specificity values were calculated at the optimal cut off points. The precision–recall curve was calculated to demonstrate the relationship between precision and recall, which provides complementary information to the ROC curve since the dataset included imbalanced classes. To address the imbalanced class data, we also calculated F_1_‐score, a measure of accuracy that considers both the precision and the recall of the test, for each model. The best F_1_‐score is 1, indicating perfect precision and recall, and the worst is 0. All the 95% confidence interval values were calculated with bootstrapping methods iterated 1000 times.^23^

## Results

### MS patient and lesion characteristics

A total of 38 patients, 12 males and 26 females, were recruited for this study. The patients averaged 55 years old (± 10.6 years). Among these patients, 15 had primary progressive MS, 10 had secondary progressive MS, and 13 had relapsing remitting MS (Table 1). Total 92 PBH lesions, 89 PGH lesions, 16 AGH lesions, 189 NBH lesions, and 113 NAWM regions were identified, with average volumes of 108.3, 66.5, 141.1, 60.6, and 120.9 mm^3^, respectively. Three of the 38 subjects had no PBH or PGH lesions.

### Histogram analysis of different MRI metrics

Distribution profiles were created using metrics derived from cMRI, DTI, DBSI, and MTR for the different MS lesion types (Fig. 2). Overall, the five lesion subtypes showed a similar hierarchical pattern regardless of the imaging metric used. Upon visual examination of the distributions, most of the imaging metrics showed a contain level of distribution differences, but no individual metrics was sufficient to discriminate lesions types using regular statistical comparisons. DNN was the better choice to recognize the patterns of such complexity in lesions.

### DNN model optimization and validation

**Figure 3:**
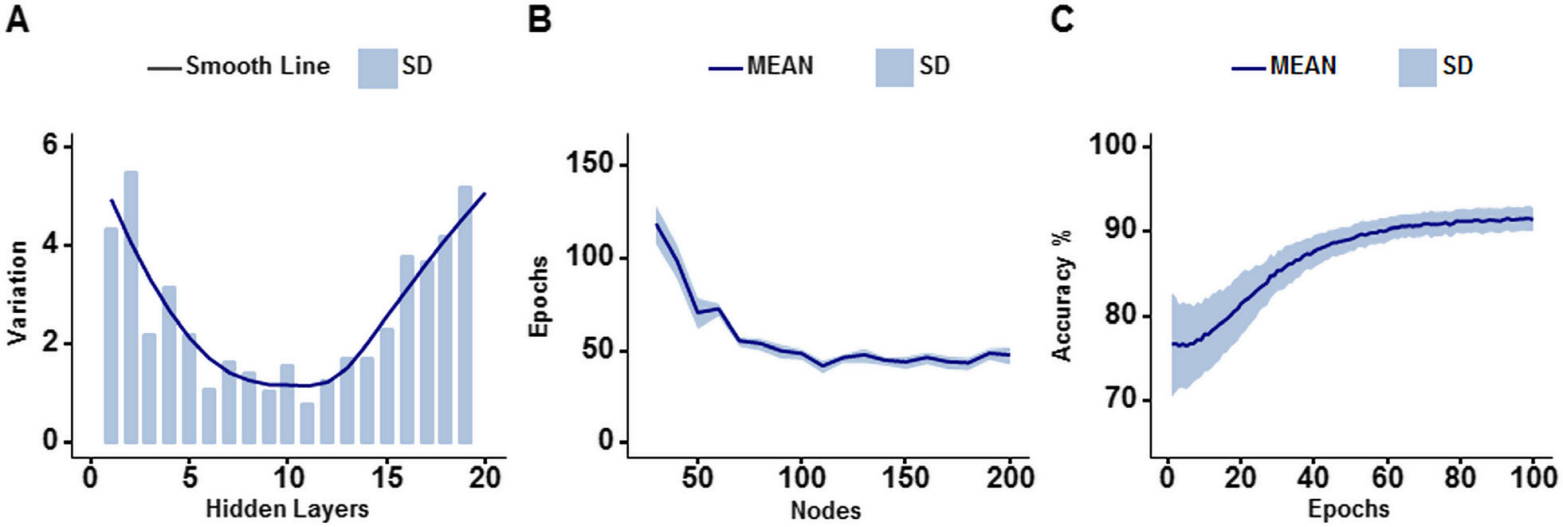
Comparison of DBSI‐DNN model with varying number of hidden layers and node count. (A) Neural nets with 1 to 20 hidden layers were tested on 70 different random states of our data for validation accuracy. About 80% of the data was used to train the DNN, 10% was used for testing and another 10% for validating. Reliability/predictability of neural networks are modelled by standard deviation. All hidden layers tested contain 100 nodes. (B) The number of epochs required for neural networks to reach 90% validation accuracy are shown. Neural networks with 10 to 200 nodes in each hidden layer were tested. All neural networks contain 10 hidden layers. Each neural network was tested via 10‐fold cross validation tests 10 times. Neural networks with 10 and 20 nodes did not attain a validation accuracy of 90% in any of its trials within 150 epochs, and therefore are not shown in the figure. (C) The optimized neural network of 10 hidden layers each containing 100 nodes was tested on 70 different random states of our data for validation accuracy. This graph shows the validation accuracies over these trials.

The optimization of DNN models were assessed by comparing overall validation accuracies of all four models. DNN with none to 11 hidden layers have smaller standard deviations than DNN with more or fewer hidden layers, indicating a better reliability (Fig. 3A). Further, optimal number of training epochs and nodes in each hidden layer were tested. DNN with 100 to 200 nodes per hidden layer was optimal, which required less than 100 training epochs to achieve 90% validation accuracy (Fig. 3B). DNN with fewer nodes per hidden layer needed an increasing number of epochs to attain 90% validation accuracy in an exponential fashion. In summary, we demonstrated that the optimal DNN structure of 10 hidden layers and 100 nodes per layer could achieve over 90% accuracy and minimal standard deviation within 100 training epochs (Fig. 3C).

### Performance and comparisons of the four DNN models

**Figure 4:**
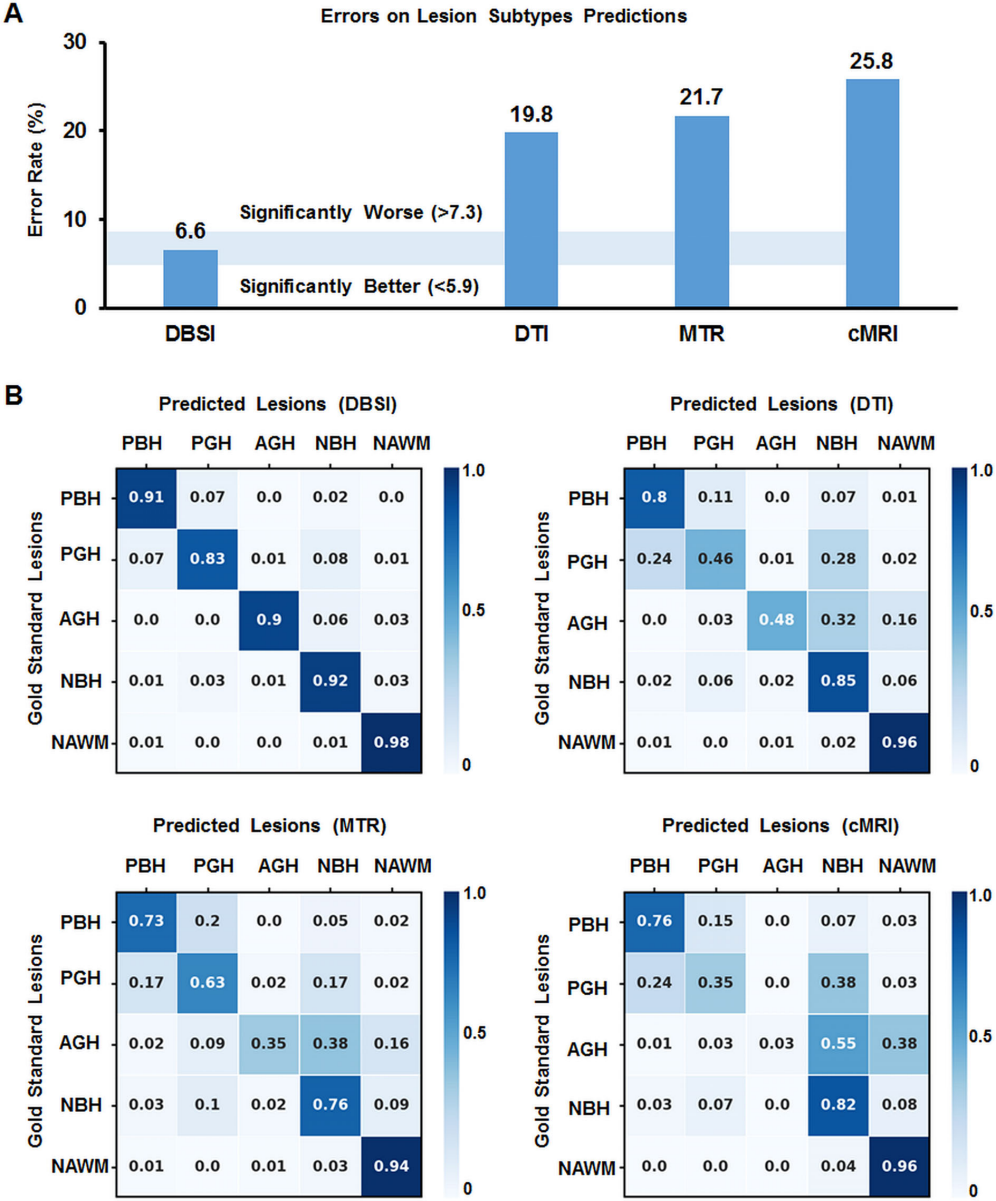
Results on lesion subtypes predictions from four different models. (A) Total error rate (1 – accuracy) on the lesion predictions from the four different models. Values outside the light blue area (5.9%–7.3%) are significantly different (95% confidence interval, using a two‐sided exact binomial test) from the DBSI‐DNN performance. (B) Confusion matrices for the predictions of the DNN models versus lesion identification using the intensity ratio method applied by an experienced neurologist (“gold standard”). Rows contain lesion classifications identified using the intensity ratio method (PBH – persistent black hole; PGH – persistent gray hole; AGH – acute gray hole; NBH – nonblack or gray hole lesion; NAWM – normal appearing white matter). Columns contain lesion classifications as predicted by different DNN models, which includes DBSI‐DNN, the DTI‐DNN model, the MTR‐DNN model and the cMRI‐DNN model.

For one independent test dataset (n = 4326), DBSI‐DNN model achieved an overall concordance with neurologist determinations of all five MS lesion subtypes with a total error rate of 6.6%, which is significantly lower than DTI‐DNN model (error rate: 19.8%), MTR‐DNN model (error rate: 21.7%), or the cMRI‐DNN model (error rate: 25.8%). We used confusion matrices to indicate the discordances between model predictions and neurologist‐determined lesion/region types derived from each model (Fig. 4). DBSI‐DNN discriminated PBH, PGH, AGH, NBH, and NAWM with positive prediction rates of 91.3%, 83.4%, 90.1%, 92.3%, and 97.9%, respectively, outperforming the other three models.

DTI‐DNN had a next‐best performance in discriminating PBH, NBH, and NAWM with rates of 80.1%, 84.8%, and 95.8%, respectively. DTI‐DNN performed worse in discriminating PGH and AGH with a 45.7% and 48.1% discrimination rates, respectively. MTR‐DNN model distinguished NAWM well with a 94% rate. PGH discrimination was only 63.3% accurate using the MTR‐DNN model. cMRI‐DNN model discriminated NBH (82.3% true positive rate) and NAWM (96.1% true positive) lesions. However, this model did not perform well on other lesion types. Specifically, PGH (35.2% true positive rate) were often incorrectly predicted to be PBH (24.4%) or NBH (37.8%) lesions. The true prediction rate of AGH was thus 3.2% with cMRI‐DNN model.

**Figure 5:**
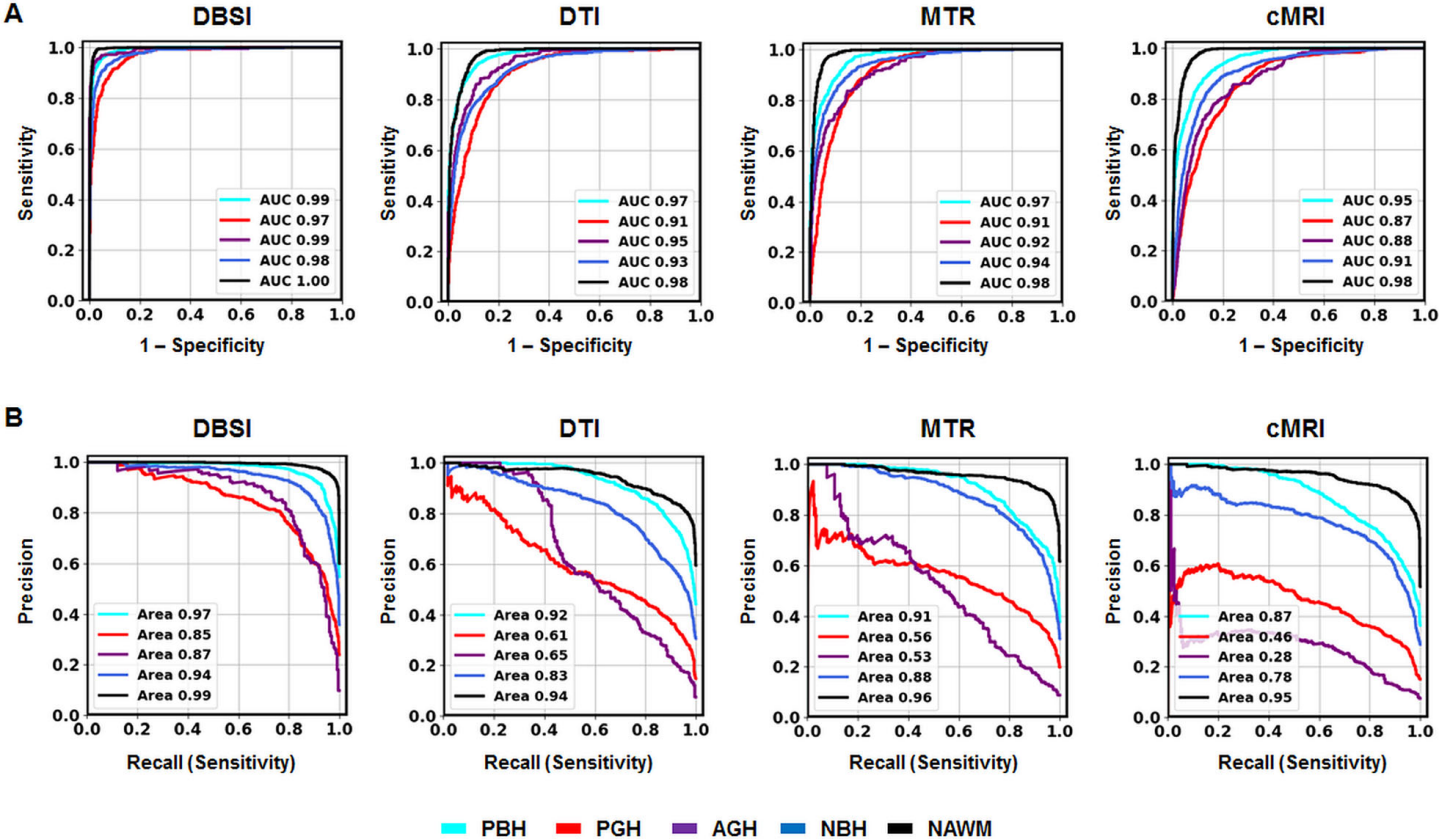
ROC and precision–recall curves. (A) ROC curves calculated on an independent test set (n = 4327) of four different models (DBSI, DTI, MTR, and cMRI). (B) Precision–recall curves calculated for DBSI, DTI, MTR, and cMRI models. DBSI showed the greatest performances on both ROC and precision–recall curves for all the five different lesions compared with other models. Class labels are as follows: Cyan, PBH. Red, PGH. Purple, AGH. Blue, NBH. Black, NAWM.

The one‐versus‐rest classification strategy was used to calculate ROC and precision–recall curves to compare the performances of each DNN model for discerning the specific lesion/tissue type. For each model, ROC (Fig. 5A) and precision–recall (Fig. 5B) curves for the five cMRI‐defined tissue types were plotted together for comparison. DBSI‐DNN demonstrated the best performance on both ROC and precision–recall analyses, with higher ROC AUC and precision–recall AUC values than any other model. DTI‐DNN, MTR‐DNN, and cMRI‐DNN ROC displayed AUC values higher than 0.860 (Fig. 5A), however, ROC analysis is insensitive to class imbalance, and could overestimate model performance. Precision–recall curves would, therefore, provide complement information to ROC. The precision–recall analyses indicated CTI‐DNN, MTR‐DNN, and cMRI DNN to perform worse than DBSI‐DNN (Fig. 5B). For example, the precision–recall AUC values for PGH and AGH in the non‐DBSI‐DNN models were all lower than 0.650 (Fig. 5B). We used bootstrap method with 1000 iterations to calculate ROC AUC, sensitivity, and specificity values for DBSI‐DNN model (summarized in Table 2).

**Table 2:** Diagnostic performances of DBSI‐DNN models.

Lesion Types	AUC (95% CI)	Sensitivity (%) (95% CI)	Specificity (%) (95% CI)	F_1_‐Score
PBH	0.991 (0.989–0.994)	95.9 (93.9–97.4)	95.0 (93.7–96.SS8)	0.923
PGH	0.977 (0.971–0.982)	92.9 (90.1–95.9)	93.4 (90.1–95.3)	0.823
AGH	0.987 (0.980–0.992)	95.1 (91.3–98.6)	95.0 (91.1–97.6)	0.887
NBH	0.981 (0.977–0.985)	93.5 (91.7–95.6)	93.1 (91.2–94.6)	0.918
NAWM	0.998 (0.997–0.998)	99.1 (97.9–99.8)	97.3 (96.3–98.4)	0.973

The 95% confidence interval values were calculated using bootstrap with 1000 iterations.

We found that DBSI‐DNN performed the best out of the four models, with a PBH F_1_‐score of 0.923, a PGH F_1_‐score of 0.823, and an AGH F_1_‐score of 0.887 (Table 2). DBSI‐DNN indicated much higher F_1_‐scores for all the MS lesions subtypes than DTI‐DNN model, MTR‐DNN model, and cMRI‐DNN model (Table S1).

## Discussion

MRI has played a vital role in the diagnosis and management of MS for decades.^24^ However, conventional T1WI and T2WI brain imaging techniques do not correlate well with MS pathologies because of the complex pathologic heterogeneity of MS lesions.^25^, ^26^ Also, conventional T1WI and T2WI imaging contrasts vary from scan to scan and are not quantitative, as they depend not only on the MR characteristics of brain tissue but also the scanner vendors, magnet strength, and pulse sequences.

Our goal is to develop a means to noninvasively evaluate the underlying pathology in living people with MS and other CNS disorders. We previously developed DBSI demonstrating its ability to quantitatively characterize CNS pathologies in postmortem MS specimens and in preclinical MS models.^11^, ^12^, ^13^, ^14^ Here, we hypothesized that DBSI‐DNN would be able to distinguish various MS lesion types.

To test our hypothesis, we compared DBSI, DTI, MTR, and conventional MRI, each in combination with optimized DNN model, in their classification accuracies on the four common MS lesion subtypes and NAWM. We found that using DBSI, T1WI, and T2WI as DNN inputs produced the most accurate classification results. Confusion matrices indicated that models trained on DBSI metrics had higher positive prediction rates, and the ROC and precision–recall curves showed that the DBSI‐DNN model had greater overall classification accuracy for each of the lesion type than DNN models based on other three commonly used imaging methods.

DBSI outperformed DTI, a widely applied imaging method for imaging CNS disorders.^27^, ^28^, ^29^ DTI’s prevalence can be attributed to its metrics’ ability to correlate with axonal injury and demyelination,^17^, ^30^, ^31^ however, this single tensor Gaussian diffusion model is inadequate for resolving coexisting complicated pathologies.^32^ DBSI adopts a novel data‐driven model that models and quantifies isotropic and anisotropic diffusion tensors within imaging voxels.^12^, ^20^, ^33^ Based on immunohistochemical data from a murine MS model and human CNS tissue specimens, restricted and nonrestricted isotropic diffusion reflects inflammatory components as well as intrinsic cells and extra‐cellular space, whereas anisotropic diffusion reflects axonal fibers.^14^, ^34^ DBSI‐derived metrics reflected specific components of MS CNS pathology, such as demyelination, edema, and increased cellularity.^13^, ^14^ DBSI outperforms conventional DTI in detecting complex MS pathologies.

MS lesion burden has often been reported as the sum of lesion volumes, but the degree of tissue destruction may vary among lesions.^35^ A prior comparison of imaging and neuropathology in over 100 MS lesions reported the degree of hypointensity to strongly associate with axonal density.^36^ In comparison to other MS lesion types, the pathologic correlation of PBH lesions contains more axon loss and extracellular matrix destruction.^37^, ^38^, ^39^ Counts and volume of PBHs positively correlated with neurological disability.^40^, ^41^ Compared to PBHs, PGHs reflect a lower degree of axonal loss. In contrast to PBH and PGH lesions, the “black” and “gray” areas of ABH and AGH lesions are more likely caused by inflammation and edema, since most ABH/AGH lesions will resolve to become isointense on T1WI within months of contrast resolution.^27^ Here we showed that DBSI metrics combined with DNN enabled accurate classification of MS lesions, which is important because different MS lesion types are associated with different clinical outcomes. A quantitative method to distinguish each MS lesion type could improve patient monitoring and potentially be useful to measure outcome in clinical trials.^36^

The relatively small number of subjects (n = 38) and the naturally heterogeneous MS lesions of our data limited the general implications of this study. However, we performed DNN analyses on 499 MS lesions, containing a total of 43,261 imaging voxels. We performed a voxel‐based computation to derive DBSI metrics, which avoids the issues concerning heterogeneity of MS lesions. The data distribution was unbalanced among different lesion and region types. Although this could compromise the performance of a DNN model, we employed precision–recall curves to provide complement ROC analyses. This study was based on data from a single institution using the same scanner. In the future, we will examine classification models across different scanner platforms and acquisition parameter variations.

## Conclusions

A DNN analysis based on DBSI (“DBSI‐DNN”) provided a 93.4% prediction accuracy in classifying MS lesions subtypes. This model outperformed DTI‐based or MTR‐based DNN models. DBSI‐DNN demonstrates great promise as a marker of lesion subtype, which is an indicator of lesion severity, particularly in relationship to axonal loss. Future additional longitudinal studies with larger cohorts, different scanners, and multiple centers are imperative to explore the possibilities of applying DBSI‐DNN on a broader scope.

## Conflicts of Interests

Anne H. Cross has performed consulting for: Biogen, Celgene, EMD Serono, Genentech/Roche, Novartis and TG Therapeutics. Robert T. Naismith has performed consulting for: Alexion, Alkermes, Biogen, Celgene, EMD Serono, Genentech, Genzyme, Novartis, TG Therapeutics, and Viela Bio. Other authors have no competing interests, financial or otherwise.

## Author Contributions

P.S., Z.Y., A.H.C., and S.‐K. S. designed the study, supervised the experiments, and wrote the manuscript. Z.Y., P.S., A.W., A.G., X.N., J.L., R.T.N., G.A., and A.H.C. performed the experimental work and/or its analysis, and refined the manuscript.

## Supporting information


**Table S1.** Diagnostic performances of DTI‐DNN, MTR‐DNN and cMRI‐DNN models.
**Figure S1.** Illustration of deep neural network. PBH, persistent black hole; PGH, persistent gray hole; ABH, accute gray hole; NBH, non‐back or gray hole; NAWM, normal appearing white matter.
