# Supplementary material for: Deep learning with diffusion basis spectrum imaging for classification of multiple sclerosis lesions
Source: Ann Clin Transl Neurol. 2020 Apr 18;7(5):695–706. doi: 10.1002/acn3.51037 (PMC7261762; doi:10.1002/acn3.51037)
Supplement: Supplementary file 1 — Table S1. Diagnostic performances of DTI‐DNN, MTR‐DNN and cMRI‐DNN models. Figure S1. Illustration of deep neural network. PBH, persistent black hole; PGH, persistent gray hole; ABH, accute gray hole; NBH, non‐back or gray hole; NAWM, normal appearing white matter. [file ACN3-7-695-s001.docx]

**Supplementary Table 1.** Diagnostic performances of DTI-DNN, MTR-DNN and cMRI-DNN models.

|  | Lesion  Types | AUC (95% CI) | Sensitivity (%)  (95% CI) | Specificity (%) (95% CI) | F_1_-Score |
| --- | --- | --- | --- | --- | --- |
| DTI-DNN | PBH | 0.967 (0.962 – 0.971) | 93.4 (88.8 – 96.0) | 87.1 (84.3 – 91.6) | 0.817 |
|  | PGH | 0.914 (0.905 – 0.923) | 91.3 (88.2 – 93.9) | 79.3 (77.2 – 82.0) | 0.529 |
|  | AGH | 0.933 (0.919 – 0.946) | 89.2 (83.6 – 94.8) | 83.0 (75.8 – 87.3) | 0.589 |
|  | NBH | 0.935 (0.927 – 0.942) | 86.2 (82.1– 90.8) | 86.0 (81.5 – 89.7) | 0.773 |
|  | NAWM | 0.981 (0.978 – 0.984) | 96.8 (95.6 – 97.7) | 92.0 (91.0 – 93.2) | 0.892 |
| MTR-DNN | PBH | 0.971 (0.967 – 0.975) | 91.9 (88.0 – 97.1) | 89.1 (83.5 – 92.7) | 0.780 |
|  | PGH | 0.914 (0.904 – 0.923) | 89.8 (84.1 – 94.5) | 78.6 (73.4 – 83.7) | 0.565 |
|  | AGH | 0.914 (0.897 – 0.929) | 83.4 (76.0 – 93.3) | 83.6 (72.3 – 91.3) | 0.443 |
|  | NBH | 0.938 (0.841 – 0.945) | 89.4 (87.0– 92.2) | 85.3 (82.5 – 87.6) | 0.762 |
|  | NAWM | 0.986 (0.983 – 0.988) | 96.6 (95.1 – 97.8) | 93.5 (92.2 – 94.8) | 0.913 |
| cMRI-DNN | PBH | 0.957 (0.951 – 0.962) | 91.3 (88.3 – 93.7) | 86.7 (84.4 – 89.4) | 0.784 |
|  | PGH | 0.865 (0.851 – 0.879) | 85.0 (79.6 – 92.6) | 73.4 (64.9 – 77.9) | 0.400 |
|  | AGH | 0.866 (0.844 – 0.886) | 80.0 (69.2 – 91.4) | 77.9 (66.3 – 91.3) | 0.058 |
|  | NBH | 0.908 (0.899 – 0.918) | 86.0 (83.6– 88.8) | 83.0 (79.8 – 84.8) | 0.736 |
|  | NAWM | 0.980 (0.977 – 0.983) | 97.0 (95.1 – 98.2) | 91.6 (90.2 – 93.4) | 0.902 |

The 95% confidence interval values were calculated using bootstrap with 1000 iterations.

**
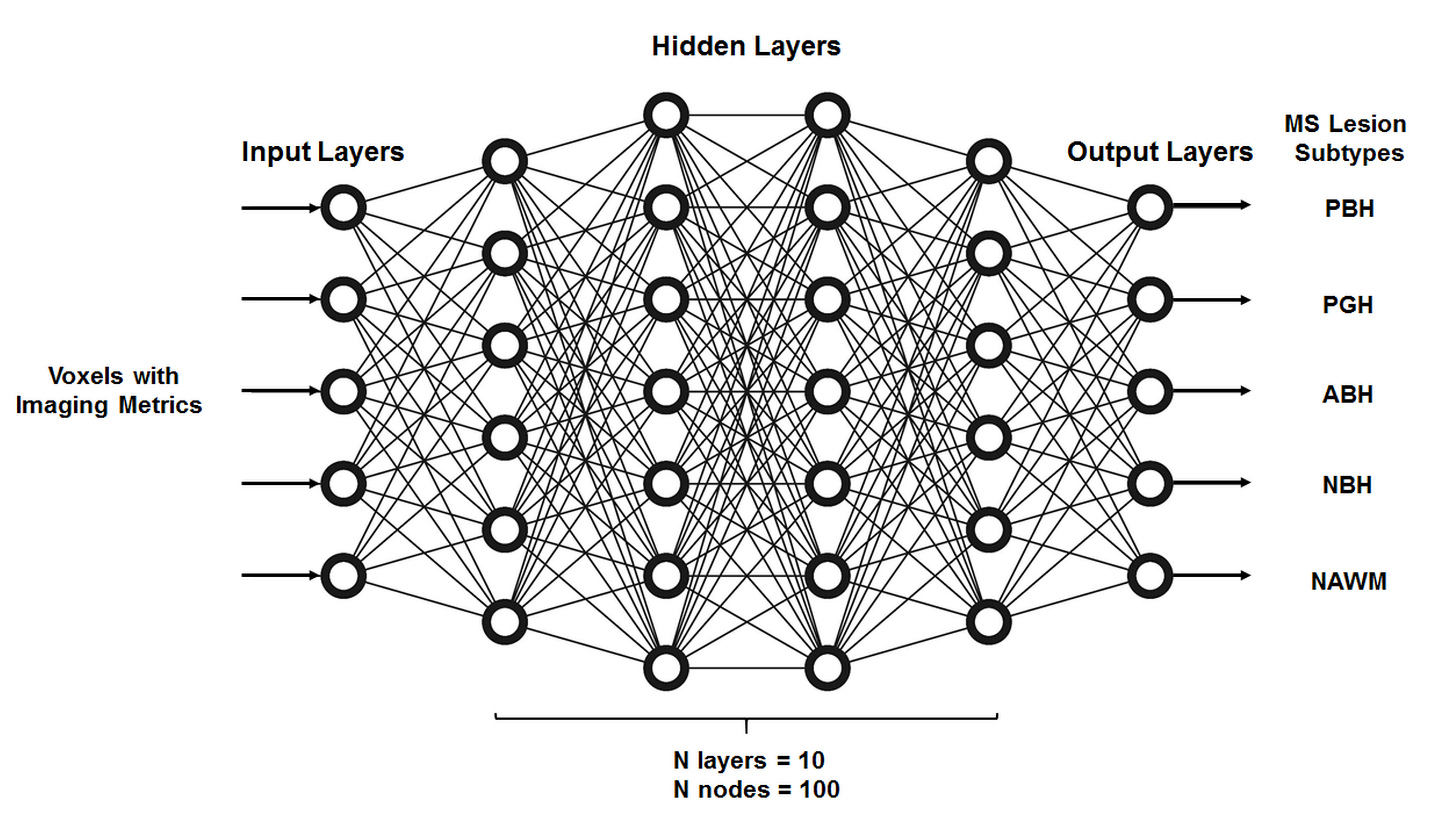
**

**Figure S1.** Illustration of deep neural network. PBH, persistent black hole; PGH, persistent gray hole; ABH, accute gray hole; NBH, non-back or gray hole; NAWM, normal appearing white matter.
